# Therapeutic targeting of both dihydroorotate dehydrogenase and nucleoside transport in *MYCN*-amplified neuroblastoma

**DOI:** 10.1038/s41419-021-04120-w

**Published:** 2021-08-30

**Authors:** Yajie Yu, Jane Ding, Shunqin Zhu, Ahmet Alptekin, Zheng Dong, Chunhong Yan, Yunhong Zha, Han-Fei Ding

**Affiliations:** 1 Institute of Neural Regeneration and Repair and Department of Neurology, The First Hospital of Yichang, Three Gorges University College of Medicine, Yichang, 443000 China; 2 Georgia Cancer Center, Medical College of Georgia, Augusta University, Augusta, Georgia 30912 USA; 3 School of Life Sciences, Southwest University, Chongqing, 400715 China; 4 Department of Cell Biology and Anatomy, Medical College of Georgia, Augusta University, Augusta, Georgia 30912 USA; 5 Charlie Norwood VA Medical Center, Augusta, GA 30904 USA; 6 Department of Biochemistry and Molecular Biology, Medical College of Georgia, Augusta University, Augusta, Georgia 30912 USA; 7 Department of Pathology, Medical College of Georgia, Augusta University, Augusta, Georgia 30912 USA

**Keywords:** Cancer metabolism, Cancer therapy

## Abstract

Metabolic reprogramming is an integral part of the growth-promoting program driven by the MYC family of oncogenes. However, this reprogramming also imposes metabolic dependencies that could be exploited therapeutically. Here we report that the pyrimidine biosynthetic enzyme dihydroorotate dehydrogenase (DHODH) is an attractive therapeutic target for *MYCN*-amplified neuroblastoma, a childhood cancer with poor prognosis. Gene expression profiling and metabolomic analysis reveal that MYCN promotes pyrimidine nucleotide production by transcriptional upregulation of DHODH and other enzymes of the pyrimidine-synthesis pathway. Genetic and pharmacological inhibition of DHODH suppresses the proliferation and tumorigenicity of *MYCN*-amplified neuroblastoma cell lines. Furthermore, we obtain evidence suggesting that serum uridine is a key factor in determining the efficacy of therapeutic agents that target DHODH. In the presence of physiological concentrations of uridine, neuroblastoma cell lines are highly resistant to DHODH inhibition. This uridine-dependent resistance to DHODH inhibitors can be abrogated by dipyridamole, an FDA-approved drug that blocks nucleoside transport. Importantly, dipyridamole synergizes with DHODH inhibition to suppress neuroblastoma growth in animal models. These findings suggest that a combination of targeting DHODH and nucleoside transport is a promising strategy to overcome intrinsic resistance to DHODH-based cancer therapeutics.

## Introduction

The ability to sustain proliferation is a fundamental feature of cancer cells [1]. In many types of cancer, this ability is acquired via constitutive or aberrant activation of the MYC family of growth-promoting oncogenic transcription factors [2–4]. Genomic amplification of *MYCN*, a member of the MYC family, is an oncogenic event in the development of high-risk neuroblastoma, a pediatric cancer of the sympathetic nervous system with poor prognosis [5–7]. In addition, aberrant MYCN activation has been implicated in the pathogenesis of adult neuroendocrine cancers, including glioblastoma, neuroendocrine prostate cancer, pancreatic cancer, and small-cell lung cancer [8].

As part of the mechanism to support its growth-promoting program, MYCN reprograms cellular metabolism to meet the biosynthetic demands associated with cell growth and proliferation. For example, MYCN transcriptionally upregulates enzymes of the serine–glycine–one-carbon (SGOC) metabolic pathway to increase the production of serine, glycine, and one-carbon units, which contribute carbon and nitrogen to purine nucleotide and thymidylate synthesis [9, 10]. However, this metabolic reprogramming, while essential to sustain cell proliferation, renders tumor cells dependent on the SGOC pathway. As a result, *MYCN*-amplified neuroblastoma cells are highly sensitive to inhibitors that block this metabolic pathway, suggesting that targeting SGOC metabolism may provide selective therapeutic benefits for patients with *MYCN*-amplified cancers [10, 11]. We reasoned that a comprehensive profiling of MYCN-activated metabolic pathways may identify new avenues that could be exploited therapeutically.

**Fig. 1:**
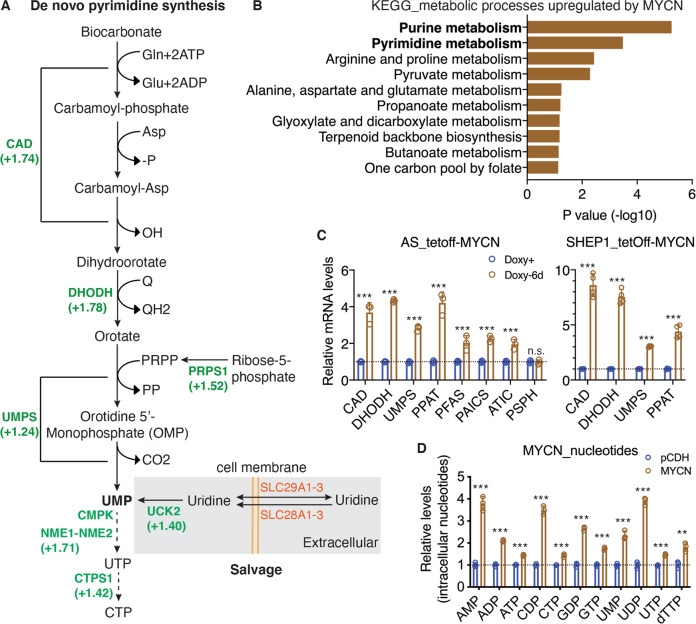
MYCN promotes nucleotide synthesis. **A** Schematic of pyrimidine biosynthesis via de novo and salvage pathways with indicated fold changes in mRNA expression of the pathway enzymes determined by microarray. **B** Bar plot of KEGG metabolic processes upregulated by MYCN via transcriptional activation. **C** qRT-PCR analysis of mRNA expression for enzymes involved in de novo nucleotide synthesis in non-*MYCN*-amplified cell lines with inducible MYCN expression in the absence of doxycycline (Doxy) for six days. Data are mean ± SD (*n* = 4). *P* values were determined by two-tailed Student’s *t*-test. ***P < 0.001, n.s., not significant. **D** Relative levels of intracellular nucleotides in non-*MYCN*-amplified SHEP1 cells with constitutive MYCN expression (MYCN) in comparison with vector-control cells (pCDH). Data are mean ± SD (*n* = 3). *P* values were determined by Welch’s *t*-test. **P < 0.01, ***P < 0.001.

Proliferating cells require increased production of pyrimidine nucleotides [12, 13]. In addition to serving as building blocks for RNA and DNA synthesis, pyrimidine nucleotides have a key role in carbohydrate and lipid metabolism, such as UDP sugars for protein glycosylation and glycogen synthesis, and CDP diacylglycerol for membrane assembly [14, 15]. In mammalian cells, pyrimidine nucleotides are produced by a combination of de novo biosynthesis and salvage (Fig. 1A). De novo biosynthesis begins with the generation of dihydroorotate from glutamine, aspartate, and bicarbonate, which is catalyzed by a trifunctional enzyme composed of carbamoyl phosphate synthase, aspartate transcarbamoylase, and dihydroorotase (CAD). The second enzyme dihydroorotate dehydrogenase (DHODH), a mitochondrial membrane protein, catalyzes the conversion of dihydroorotate to orotic acid, which, in turn, is converted into uridine monophosphate (UMP) by a bifunctional protein, UMP synthetase (UMPS). UMP is the precursor for all other pyrimidine nucleotides required for RNA and DNA biosynthesis, as well as for carbohydrate and lipid metabolism. The salvage pathway provides substrates for pyrimidine nucleotide production via two routes, recycling UMP and CMP derived from intracellular RNA degradation and importing nucleosides (uridine and cytidine) from the bloodstream. Uridine and cytidine are converted into UMP and CMP, respectively, by uridine–cytidine kinase (UCK). The uridine concentrations in the human plasma or serum are in the range of 5–20 µM [16, 17], which are at least an order of magnitude higher than the plasma concentrations of other pyrimidines, indicating that uridine is the dominant circulatory nucleoside to support cellular demands of pyrimidine nucleotides via salvage [15]. The SLC28 family of concentrative nucleoside transporters and the SLC29 family of equilibrative nucleoside transporters are primarily responsible for the uptake of nucleosides by mammalian cells [18, 19].

In this study, we investigated the role of MYCN in reprogramming pyrimidine synthesis, with a focus on its therapeutic potential. We identified DHODH as an effective drug target in *MYCN*-amplified neuroblastoma cell lines and mouse neuroblastoma models. Moreover, we showed that blocking nucleoside transport increased the efficacy of DHODH inhibitors in the presence of physiological concentrations of uridine, suggesting a therapeutic strategy to overcome plasma uridine-dependent resistance to DHODH-targeting drugs in cancer patients.

## Materials and methods

### Cell lines and culture

Neuroblastoma cell lines BE(2)-C (CRL-2268), SK-N-AS (CRL-2137), and SK-N-DZ (CRL-2149) were obtained from ATCC (Manassas, VA), LA1-55n (06041203) from Millipore Sigma (St. Louis, MO), and LA-N-5, LA-N-6, and SMS-KCNR from Childhood Cancer Repository (Texas Tech University Health Sciences Center). IMR5 was a gift from J. Cowell (Augusta University) and SHEP1 from V.P. Opipari (University of Michigan). All cell lines from commercial sources and cell-line repositories had been authenticated using short tandem-repeat profiling, and upon receiving, large frozen stocks were made to ensure against contaminations by other cell lines. IMR5 was verified as a MYCN-amplified neuroblastoma cell line by high-level nuclear expression of MYCN and the specific neuroblastoma marker PHOX2B [20, 21]. All cell lines were used within 10 passages after reviving from frozen stocks and were free of *mycoplasma* contamination as determined by a LookOut Mycoplasma PCR kit (Sigma-Aldrich) and DAPI staining every three months. SHEP1 and SK-N-AS were cultured in DMEM (HyClone SH30022), BE(2)-C in DME/F-12 1:1 (HyClone SH30023), and all other cell lines in RPMI 1640 (HyClone SH30027). All culture media were obtained from Thermo Fisher Scientific (Hampton, NH) and supplemented with 10% or 50% FBS (Atlanta Biologicals S11050, Flowery Branch, GA). For dialyzed FBS preparation, FBS in dialysis tubing 3500 MWCO (Thermo Fisher Scientific 68035) was dialyzed against phosphate-buffered saline (PBS) overnight and the dialyzed FBS was sterilized by filtration through a 0.22-µm filter unit (Millipore Sigma S2GPU01RE). Cell viability and proliferation were determined by the trypan blue exclusion assay. Cell images were acquired using an EVOS FL imaging system (Thermo Fisher Scientific).

### Patient data

Analyses of Kaplan–Meier survival and neuroblastoma stages based on gene expression were conducted online using the R2 Platform (https://hgserver1.amc.nl/cgi-bin/r2/main.cgi) and the neuroblastoma datasets SEQC-498, NRC-283, and Versteeg-88 [22–24]. The resulting figures and *P* values were downloaded. Box plots of DHODH mRNA expression in relation to neuroblastoma-risk groups and MYCN-amplification status were generated using the box-and-whiskers (Tukey) plot of GraphPad Prism 9.0d for Mac (GraphPad Software, San Diego, CA 92108).

### Overexpression and knockdown

Non-*MYCN*-amplified neuroblastoma cell lines SHEP1 and SK-N-AS with inducible MYCN expression in the absence of doxycycline (tetoff-MYCN) or with constitutive MYCN expression (pCDH-MYCN) were generated as described previously [9]. Lentiviral shRNA constructs shDHODH-21 (TRCN0000025821), shDHODH-32 (TRCN0000025832), shDHODH-68 (TRCN0000025868), shMYCN-94 (TRCN0000020694), and shMYCN-95 (TRCN0000020695) were obtained from Millipore Sigma. Lentiviruses for shRNA expression were produced in 293FT cells using the packaging plasmids pLP1, pLP2, and pLP/VSVG (Thermo Fisher Scientific K497500), and lentiviral infections of cells were conducted according to standard procedures.

### Microarray

Total RNA was isolated using TRIzol (Thermo Fisher Scientific 15596026) from three biological replicates of SK-N-AS-tetoff-MYCN cultured in the presence or absence of 1 µg/ml doxycycline for six days. Affymetrix microarray was performed using the Human Gene 2.0 ST microarray chip (Affymetrix, Santa Clara, CA). Data were normalized, significance determined by ANOVA, and fold change calculated with the Partek Genomics Suite. Gene Ontology (GO) analysis by DAVID [25] and Gene Set Enrichment Analysis (GSEA) [26] were performed as described [9]. The NCBI Gene Expression Omnibus (GEO) accession number for the microarray data is GSE164309.

### Quantitative reverse-transcription PCR (qRT-PCR)

Total RNA was isolated using TRIzol, followed by reverse transcription using an iScript Advanced cDNA Synthesis Kit (Bio-Rad 172-5038, Hercules, CA). qRT-PCR was performed using a 2X SYBR green qPCR master mix (Bimake B21202, Houston, TX) on an iQ5 real-time PCR system (Bio-Rad) with gene-specific primers (Table S1). All samples were normalized to β2 microglobulin (B2M) mRNA levels.

### Immunoblotting

Proteins (20–50 µg) were separated on SDS-PAGE, transferred to nitrocellulose membranes (926-31092, LI-COR Biosciences, Lincoln, NE), and probed with the following primary antibodies: mouse anti-DHODH, (E8, 1:1000, sc-166348, Santa Cruz Biotech, Dallas, TX), mouse anti-MYCN (NCM II 100, 1:200, Millipore OP13), rabbit anti-GAPDH (FL-335, 1:1000, sc-25778, Santa Cruz Biotech), and mouse anti-α-tubulin (B-5-1-2, 1:4000, Sigma-Aldrich). Horseradish peroxidase-conjugated goat anti-mouse (sc-2005, Santa Cruz Biotech) and anti-rabbit IgG (sc2004, Santa Cruz Biotech) were used as secondary antibodies. Proteins were visualized and quantified using a Clarity Western ECL Kit (Bio-Rad) and ImageJ (version 1.53a). Films were exposed for various times for quantification of target proteins within their linear range of detection.

### Intracellular nucleotide analysis

SHEP1-pCDH and SHEP1-pCDH-MYCN cells (~80% confluency) were washed with 5% mannitol and extracted with HPLC-grade methanol (Thermo Fisher Scientific A452-4) containing internal standards (H3304-1002, Human Metabolome Technologies, Inc. HMT, Tsuruoka, Japan). Extracts were collected and centrifuged. The resulting supernatants were filtered through a centrifuge filter unit provided by HMT and dried by vacuum centrifugation. Extracted metabolites were analyzed by capillary electrophoresis time-of-flight mass spectrometry (CE-TOFMS) at HMT [27, 28]. Peaks were annotated with putative metabolites from the HMT metabolite database based on their MTs/RTs and *m*/*z* values determined by TOFMS. The tolerance range for the peak annotation was configured at ±0.5 min for MT and ±10 ppm for *m/z*. Peak areas were normalized against the internal standards and then the cell number. Three biological replicate samples (~10^6^ cells/sample) were analyzed for each cell type and *p* values were calculated by Welch’s *t*-test.

### Chromatin immunoprecipitation and quantitative PCR (ChIP-qPCR)

ChIP was performed using *MYCN*-amplified neuroblastoma BE(2)-C cells as described [10]. For each antibody, ~4 × 10^7^ cells were used. Cross-linked chromatin was sheared through sonication (Thermo Fisher Scientific Model 150E) and immunoprecipitated using Dynabeads protein G (Thermo Fisher Scientific 10004D) coated with ChIP-grade mouse anti-MYCN (B8.4.B, sc-53993, Santa Cruz Biotech) or control mouse IgG (sc-2025, Santa Cruz Biotech). For qPCR, two independent ChIP samples were analyzed using the following ChIP-qPCR primers: DHODH-274 forward, TGCGGTAGTACGGACAAACAC; reverse, GGGCAGAAGGGTTGAGAGGAC; DHODH + 179 forward, GGTCTCCTGCAAATGCTCGTG; reverse, GTTCACTAAGCACCCCTGTGC; DHODH + 3965 forward, CAGAGTCATTAAGCACTGGTG; reverse, CACTTCTGCCTACACACCCTC; and MDM2p forward, AGCCTTTGTGCGGTTCGTG; reverse, CCCCCGTGACCTTTACCCTG. The number associated with each primer set indicates the position of the forward primer relative to its target gene transcription-start site (TSS, +1). Data on MYC binding to the human *DHODH* locus were obtained from ENCODE Transcription Factor Binding tracks on UCSC genome browser [29, 30].

### Bromodeoxyuridine (BrdU) immunofluorescence

Cells were grown on coverslips, and BrdU (Sigma-Aldrich B5002) was added to the culture media at the final concentration of 10 µg/ml for 30 min. Cells were fixed in 4% paraformaldehyde and permeabilized with 0.3% Triton X-100. After blocking with 10% goat serum, the coverslips were incubated with rat anti-BrdU (1:200, ab6326, Abcam, Cambridge, MA) and then with the secondary antibody Alexa Fluor 488 goat anti-rat IgG (H + L, 1:800, Thermo Fisher Scientific A-11006). Nuclei were stained with DAPI. Fluorescent images were captured with a Nikon microscope Eclipse 80i (Nikon Instruments Inc., Melville, NY). DAPI- and BrdU-positive cells were counted from randomly selected 200x fields from three independent experiments, and the percentage of BrdU-positive cells was determined.

### Soft-agar clonogenic assay

Cells (~1000/well) were mixed with 0.3% Noble agar in DMEM culture media and plated onto six-well plates containing a solidified bottom layer (0.6% Noble agar in DMEM culture media). After 14 days, colonies were stained with 5 mg/ml MTT (Sigma-Aldrich M5655) in PBS, photographed, and counted.

### Inhibitor assays

Brequinar sodium (BRQ, Tocris 96201-88-6, Minneapolis, MN), GSK983 (Sigma-Aldrich SML1824), leflunomide (Selleckchem 75706-12-6, Houston, TX), dipyridamole (Sigma-Aldrich D9766), and MLN8237 (Selleckchem S1133) were dissolved in dimethyl sulfoxide (DMSO, Thermo Fisher Scientific BP231-100), and stock solutions were aliquoted and stored at −80°C until use.

For DHODH-inhibition studies, cells were treated with DMSO, BRQ, GSK983, and/or dipyridamole in the presence or absence of uridine (Sigma-Aldrich U3003), orotic acid (Sigma-Aldrich 02750), or nucleoside mix (Millipore Sigma EmbryoMax nucleosides ES-008-D) at the indicated concentrations and times, and the number of viable cells was determined by the trypan blue exclusion assay. Inhibitor dose–response curves were fitted with the four-parameter equation “log (inhibitor) vs. response – variable slope” and IC_50_ values were determined using GraphPad Prism 9.0d for Mac. Cells were also collected for immunoblotting and qRT-PCR analyses of the expression of pyrimidine-synthesis enzymes.

For Aurora-A-inhibition study, cells were treated with DMSO or MLN8237 at 1.0 µM for 24, 48, or 72 h and collected for immunoblot and qRT-PCR analyses of MYCN and DHOHD expression.

### In vivo studies

NOD.SCID male and female mice of 6-week-old (NOD.Cg-Prkdc^scid^/J, Jackson Laboratory 001303, Bar Harbor, ME) were randomly assigned to different groups (*n* = 5 per group). For shDHODH study, 3 × 10^6^ BE(2)-C cells expressing either shGFP or shDHODH-68 were suspended in 100 µl of Hanks’ Balanced Salt Solution (HBSS, Gibco 14170-112, Thermo Fisher Scientific) and injected subcutaneously into the flanks of the mice (1 site per mouse). For drug-treatment studies, BE(2)-C and SMS-KCNR cells in 100 µl of HBSS were injected subcutaneously into both sides of the flank (two sites per mouse) at 3 × 10^6^ cells per injection site. Following injection, mice were randomly assigned to control and treatment groups. Tumor volume was measured every other day using a digital caliper and estimated using the equation *V* = (*L* × *W*^2^)/2. Animals were euthanized when their tumors reach ~2.0 cm in diameter.

For in vivo BRQ treatment, BRQ at 40 mg/ml in H_2_O was diluted to 30% PEG-400 (Sigma-Aldrich 06855) in PBS. Mice were treated with vehicle (30% PEG-400 in PBS) or BRQ at 50 mg/kg in a final volume of 100 µl every three days by intraperitoneal injection for 21–24 days (until all the mice in the vehicle group were sacrificed when their tumors reached ~2.0 cm in diameter). For BRQ treatment of established BE(2)-C xenografts, mice were treated with vehicle (30% PEG-400 in PBS) or BRQ at 25 mg/kg in a final volume of 100 µl every other day by intraperitoneal injection for 35 days. For combination treatment, dipyridamole at 25, 50, or 100 mg/ml DMSO was diluted with 50% PEG-400 in PBS at the ratio of 1:1.5 to a final concentration of 10, 20 or 40 mg/ml (40% DMSO–30% PEG-400 in PBS). Mice were treated by intraperitoneal injection for 22 days with vehicle (40% DMSO–30% PEG-400 in PBS) daily, BRQ at 25 mg/kg (30% PEG-400 in PBS) every other day, dipyridamole at 50, 100, or 200 mg/kg (40% DMSO–30% PEG-400 in PBS) daily, or BRQ (every other day) in combination with dipyridamole at 50, 100, or 200 mg/kg (daily).

*TH-MYCN* transgenic mice (129×1/SvJ-Tg(TH-MYCN)41Waw/Nci) were obtained from the NCI Mouse Repository (Frederick, MD). Male and female *TH-MYCN* mice of 30-day-old were randomly assigned to 4 groups and treated by intraperitoneal injection for 30 days with vehicle daily, BRQ at 25 mg/kg every other day, dipyridamole at 200 mg/kg daily, or BRQ (every other day) in combination with dipyridamole at 200 mg/kg (daily) as described above. All mice were monitored for tumor progression, until euthanasia was required.

The animal experiments were approved by the Institutional Animal Care and Use Committee of Medical College of Georgia, Augusta University.

### Statistics

For all cell-based studies, two to four independent experiments were performed with each cell line and three to four technical replicates per experiment (n values in the corresponding figure legends). Quantitative data are presented as mean ± SD and were analyzed for statistical significance by unpaired, two-tailed Student’s *t*-test (two groups) or two-way ANOVA (more than two groups). For mouse studies, *P* values were determined by the log-rank (Mantel–Cox) test for mouse survival. All quantitative data showed apparent normal distribution and equal variance. Unless otherwise stated, all statistical analyses were performed using GraphPad Prism 9.0d for Mac.

## Results

### MYCN transcriptionally enhances nucleotide synthesis

To identify metabolic pathways that are activated at the transcription level by MYCN, we performed microarray gene expression profiling of MYCN-responsive genes, using the non-*MYCN*-amplified neuroblastoma cell line SK-N-AS with inducible expression of MYCN in the absence of doxycycline [10]. A total of 1370 MYCN-responsive genes (≥±1.50-fold, *p* < 0.05) were identified, with 599 genes being upregulated and 771 genes downregulated (Table S2). Pathway analysis using the Kyoto Encyclopedia of Genes and Genomes (KEGG) pathway database [31] revealed that MYCN-upregulated genes were significantly enriched for KEGG pathways associated with purine and pyrimidine metabolism (Fig. 1B and Table S3). We obtained essentially the same results with gene set enrichment analysis (GSEA), which showed upregulation of gene sets involved in pyrimidine and purine biosynthesis and metabolism (Fig. S1A–E).

We confirmed the microarray data by qRT-PCR (Fig. 1C), which revealed that MYCN overexpression in non-*MYCN*-amplified neuroblastoma SK-N-AS and SHEP1 cells significantly increased mRNA expression of genes encoding enzymes responsible for de novo synthesis of purine (PPAT, PFAS, PAICS, and ATIC, see also Fig. S1C) and pyrimidine (CAD, DHODH, and UMPS) nucleotides. To determine the functional significance of their upregulation by MYCN, we analyzed the intracellular nucleotide pools in SHEP1 cells with or without MYCN overexpression. Compared with vector-control cells (pCDH), MYCN-overexpressing cells (MYCN) showed a significant increase in the levels of purine and pyrimidine nucleotides (Fig. 1D). Together, these findings suggest that MYCN increases the production of purine and pyrimidine nucleotides by transcriptional upregulation of their biosynthetic enzymes.

### DHODH is a transcriptional target of MYCN and is expressed at higher levels in *MYCN*-amplified neuroblastoma tumors

**Fig. 2:**
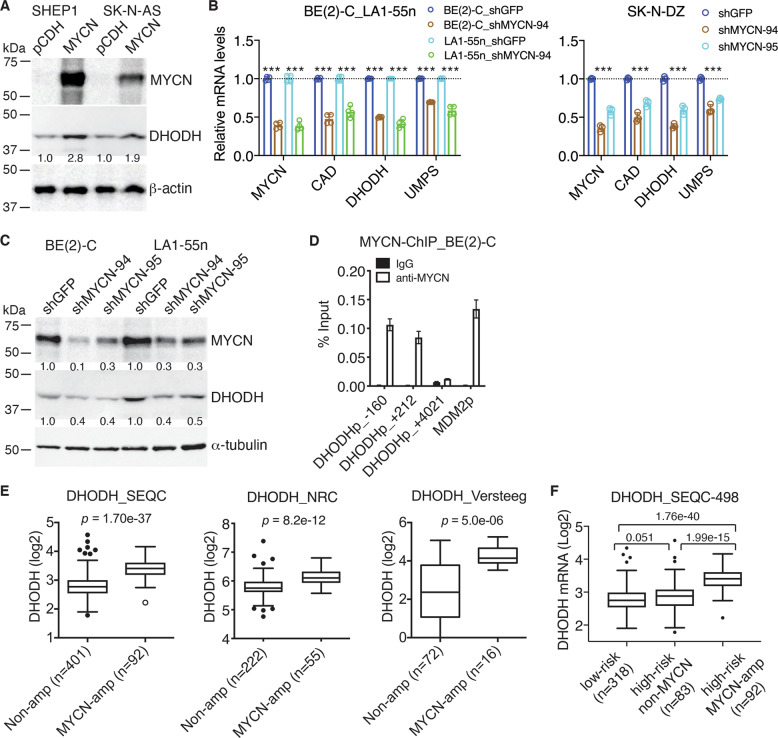
DHODH is a direct transcriptional target of MYCN in neuroblastoma. **A** Immunoblot analysis of DHODH expression in non-MYCN-amplified neuroblastoma cell lines infected with vector (pCDH) or MYCN-expressing (MYCN) lentiviruses. DHODH levels were quantified against β-actin. Data are representative of two independent experiments. **B** qRT-PCR analysis of pyrimidine-pathway enzyme expression in *MYCN*-amplified cell lines with MYCN knockdown. Data are mean ± SD (*n* = 4). *P* values were determined by two-tailed Student’s *t*-test. ***P < 0.001. **C** Immunoblot analysis of DHODH expression in *MYCN*-amplified cell lines with MYCN knockdown. DHODH levels were quantified against α-tubulin. Data are representative of three independent experiments. **D** ChIP-qPCR showing endogenous MYCN binding to the *DHODH* promoter and 1^st^ intron in *MYCN*-amplified BE(2)-C cells. The *MDM2* promoter was used as a positive control. Data are mean ± SD (*n* = 3). **E, F** Box plots of DHODH mRNA expression in relation to *MYCN*-amplification status (**E**) and neuroblastoma-risk groups (**F**) using the SEQC, NRC, and Versteeg datasets. Data were analyzed by unpaired, two-tailed Student’s *t*-test with *P* values indicated.

We next focused our investigation on the pyrimidine biosynthetic enzyme DHODH, given its potential as a therapeutic target [32–36]. Consistent with the transcription data shown above (Fig. 1A–C), overexpression of MYCN in non-*MYCN*-amplified SHEP1 and SK-N-AS cell lines increased DHODH protein expression (Fig. 2A). Conversely, knockdown of MYCN expression in *MYCN*-amplified BE(2)-C, LA1-55n, and SK-N-DZ cell lines by two independent shRNA constructs reduced DHODH mRNA (Fig. 2B) and protein expression (Fig. 2C). In addition, we treated BE(2)-C and LA1-55n cells with the Aurora-A-specific inhibitor MLN8237, which has been shown to disrupt the Aurora A–MYCN complex, leading to MYCN degradation [37]. Consistent with the previous report [37], treatment with MLN8237 markedly reduced MYCN protein levels (Fig. S2A) but had no consistent effect on *MYCN* mRNA expression (Fig. S2B). MLN8237 treatment also significantly inhibited the expression of DHODH and other pyrimidine-synthesis enzymes (Fig. S2A, B). Collectively, these data indicate that MYCN is a transcriptional activator of DHODH expression and is essential for maintaining DHODH expression in *MYCN*-amplified neuroblastoma cell lines.

We then asked whether MYCN directly targets *DHODH* for transcriptional activation. We found the presence of the canonical MYC-binding E-box sequence CACGTG in the *DHODH* promoter region (–160, +1 being the transcription-start site), first intron (+212), and second intron (+4021). In addition, an examination of ENCODE data on chromatin immunoprecipitation and massively parallel DNA sequencing (ChIP-seq) of transcription factors [29, 30] revealed specific MYC binding to the *DHODH* promoter and first intron, as well as the second intron (Fig. S2C). Our ChIP-qPCR analysis in *MYCN-*amplified BE(2)-C cells showed significant levels of endogenous MYCN in the E-box-containing regions in the *DHODH* promoter and first intron (Fig. 2D). Together, these data indicate that *DHODH* is a direct transcriptional target gene of MYCN in neuroblastoma cells.

To assess the clinical relevance of DHODH upregulation by MYCN, we analyzed gene expression profiling data from three independent neuroblastoma patient cohorts (SEQC, NRC, and VERSTEEG) [22–24]. DHODH mRNA expression is significantly higher in neuroblastoma tumors with *MYCN* amplification than those without (Fig. 2E). More specifically, high-risk tumors carrying *MYCN* amplification express significantly higher levels of DHODH mRNA relative to low-risk tumors and high-risk tumors without *MYCN* amplification (Fig. 2F). Furthermore, increased DHODH mRNA expression is significantly associated with reduced survival in neuroblastoma patients (Fig. S2D) and advanced stages of the disease (Fig. S2E). Collectively, these findings suggest that DHODH is an important downstream target of MYCN in driving high-risk neuroblastoma pathogenesis and progression.

### High DHODH expression is required for the proliferation and tumorigenicity of *MYCN*-amplified neuroblastoma cell lines

**Fig. 3:**
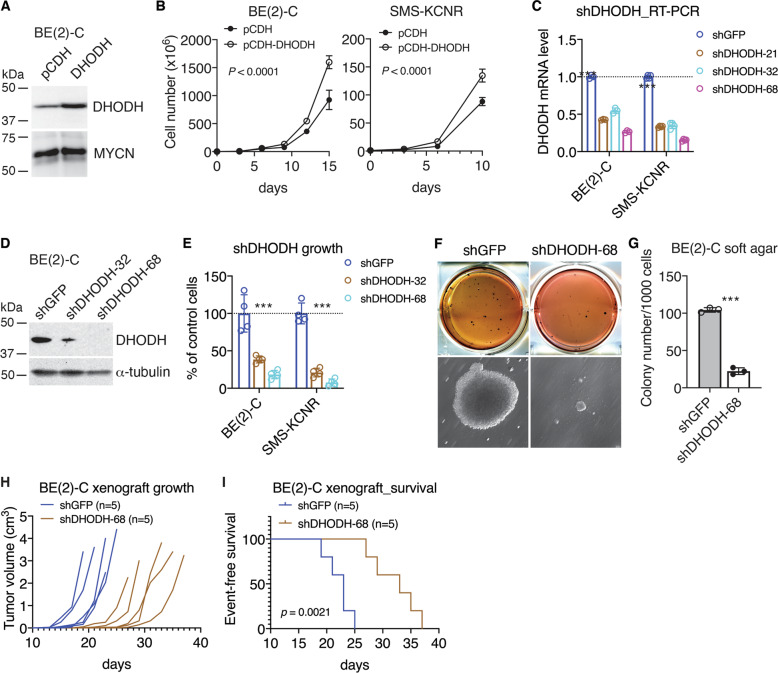
High DHODH expression is required for neuroblastoma cell proliferation and tumorigenicity. **A** Immunoblot analysis of DHODH expression in vector control and DHODH-overexpressing BE(2)-C cells. MYCN levels are shown as loading control. **B** Cell-growth assay of vector control and DHODH-overexpressing *MYCN*-amplified cell lines. Data are mean ± SD (*n* = 4). *P* values were determined by ANOVA. **C, D** qRT-PCR (**C**) and immunoblot (**D**) analyses of DHODH expression in *MYCN*-amplified cell lines expressing shRNA to GFP or DHODH. Data in (**C**) are mean ± SD (*n* = 4). *P* values were determined by two-tailed Student’s *t*-test. ***P < 0.001. α-tubulin levels are shown as loading control (**D**). **E** Cell-growth assay of *MYCN*-amplified cell lines without (shGFP) or with DHODH knockdown (shDHODH) for four days. Data are mean ± SD (*n* = 4). *P* values were determined by two-tailed Student’s *t*-test. ***P < 0.001. **F, G** Soft-agar clonogenic assay of BE(2)-C cells without (shGFP) or with DHODH knockdown (shDHODH). Data in (**G**) are mean ± SD (*n* = 3). *P* values were determined by two-tailed Student’s *t*-test. ***P < 0.001. **H, I** Tumor growth (**H**) and event-free survival (**I**) curves for mice bearing xenografts of BE(2)-C cells without (shGFP) or with DHODH knockdown (shDHODH). Log-rank test *P* value is indicated (**I**).

To investigate the functional significance of increased DHODH expression, we generated BE(2)-C and SMS-KCNR cell lines with DHODH overexpression (Fig. 3A). DHODH overexpression significantly, albeit modestly, enhanced cell proliferation (Fig. 3B). To assess the effect of DHODH knockdown, we tested lentiviral constructs expressing shRNA sequences against different regions of the DHODH gene by qRT-PCR (Fig. 3C) and selected shDHODH-32 and shDHODH-68 for their ability to silence DHODH protein expression (Fig. 3D). DHODH knockdown significantly inhibited the growth of BE(2)-C and SMS-KCNR cells (Fig. 3E and Fig. S3A) and reduced the number of BrdU-positive, proliferating cells (Fig. S3B, C). Similarly, DHODH knockdown inhibited the growth of non-MYCN-amplified neuroblastoma cell lines SHEP1 and SK-N-AS (Fig. S3D–F). In line with the cell-growth data, knockdown of DHODH expression markedly inhibited the anchorage-independent growth of BE(2)-C cells in soft agar (Fig. 3F, G), slowed the growth of BE(2)-C xenografts in immunodeficient mice (Fig. 3H and Fig. S3G), and prolonged the survival of tumor-bearing mice (Fig. 3I). Together, these data demonstrate an essential role of DHOHD in maintaining the proliferative state of neuroblastoma cells in vitro and in vivo.

### DHODH is a therapeutic target in neuroblastoma

We have shown previously that treatment with the DHODH inhibitor leflunomide reduces neuroblastoma cell survival in culture and tumorigenicity in immunodeficient mice [32]. However, neuroblastoma cell lines are relatively resistant to leflunomide, and only at ~100 µM did we consistently observe a significant growth-inhibitory effect on a panel of neuroblastoma cell lines (Fig. S4A). To further explore DHODH as a therapeutic target in neuroblastoma, we tested additional small-molecule inhibitors of DHODH, focusing on brequinar sodium (BRQ) and GSK983. BRQ was originally developed by DuPont (DUP 785; NSC 368390) as an anticancer drug [38] and was subsequently shown to be a potent and selective inhibitor of DHOHD [39, 40]. GSK983 was originally developed by GlaxoSmithKline as a broad-spectrum antiviral agent [41, 42] and was recently identified as a specific DHODH inhibitor [43].

**Fig. 4:**
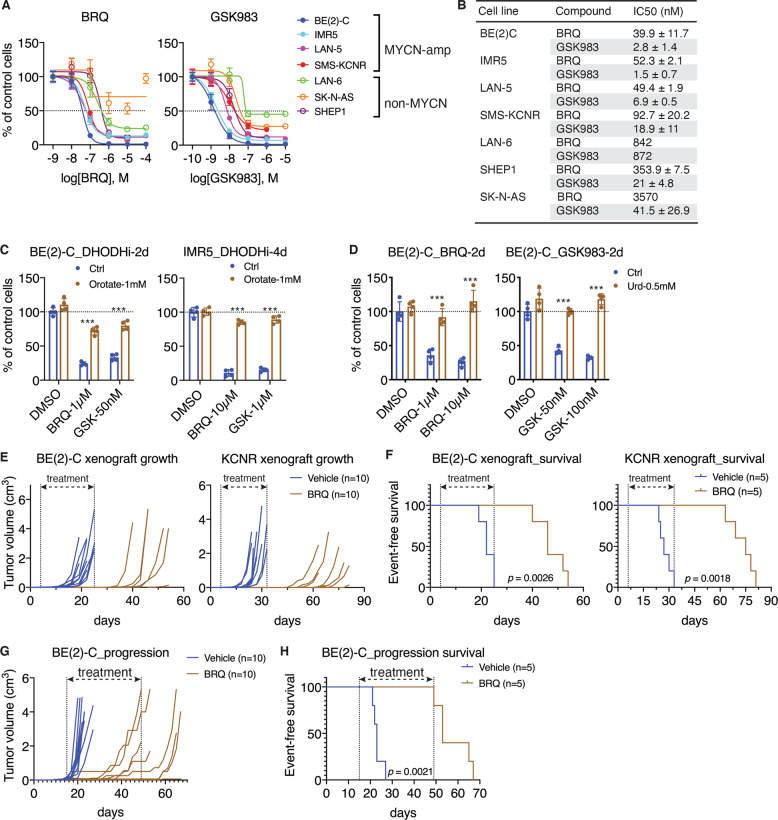
DHODH is a therapeutic target in neuroblastoma. **A** Dose–response curves for BRQ and GSK983 in indicated human neuroblastoma cell lines following four days of treatment. Data are mean ± SD (*n* = 4). **B** IC50 values for BRQ and GSK983 in the indicated cell lines following four days of treatment. Data are mean ± SD from at least two independent experiments (four technical replicates for each cell line per experiment). **C, D** Cell-growth assays of indicated neuroblastoma cell lines treated with DMSO (vehicle control), BRQ, or GSK983 in the absence (Ctrl) or presence of supplemental orotate (**C**) or uridine (Urd, **D**) for two or four days. Data are mean ± SD (*n* = 4). *P* values were determined by two-tailed Student’s *t*-test. ***P < 0.001. **E, F** Tumor-growth (**E**) and event-free survival (**F**) curves for mice-bearing xenografts of BE(2)-C or SMS-KCNR cells treated with vehicle or BRQ at 50 mg/kg every three days. Log-rank test *P* values are indicated. **G, H** Tumor growth (**G**) and event-free survival (**H**) of mice bearing established xenografts of BE(2)-C cells treated with vehicle or BRQ at 25 mg/kg every other day. Log-rank test *P* value is indicated.

We examined the effect of BRQ and GSK983 on cell proliferation using a panel of seven neuroblastoma cell lines with or without *MYCN* amplification. Both BRQ and GSK983 displayed a marked inhibitory effect on the proliferation of neuroblastoma cell lines cultured in standard media containing 10% FBS (Fig. 4A), showing IC_50_ values in the range of nanomoles to low micromoles (Fig. 4B). Overall, *MYCN*-amplified cell lines were significantly more sensitive to BRQ and GSK983 compared with non-*MYCN*-amplified cell lines, as indicated by a decrease of ~10-fold or more in IC_50_ values (Fig. 4B). The growth-inhibitory effect of both BRQ and GSK983 could be reversed by supplemental orotate (Fig. 4C), the product of the reaction catalyzed by DHODH (Fig. 1A), or by uridine (Fig. 4D), which can be used to generate pyrimidine nucleotides via the salvage pathway (Fig. 1A). These findings indicate that DHODH is the primary target of BRQ and GSK983 in blocking the proliferation of neuroblastoma cell lines. Moreover, treatment with BRQ and GSK983 had no effect on the expression of DHODH or other enzymes of the pyrimidine-synthesis pathway in the absence or presence of MYCN overexpression (Fig. S4B), providing further evidence that these inhibitors repressed neuroblastoma cell proliferation by inhibiting DHOHD enzyme activity but not by regulating its expression.

We next evaluated the antitumor activity of BRQ, which is water-soluble, in mouse xenograft models established with *MYCN*-amplified BE(2)-C and SMS-KCNR cells. On day 4 (BE(2)-C) or day 6 (SMS-KCNR) post inoculation, mice were randomly assigned to two groups and treated with vehicle or 50 mg/kg (body weight) of BRQ by intraperitoneal injection once every three days for 21 (BE(2)-C) or 30 days (SMS-KCNR), when all the vehicle-control mice were sacrificed due to tumor progression. During treatment, mice of the BRQ group displayed no detectable xenograft growth, whereas the vehicle group showed fast-growing xenografts (Fig. 4E and Fig. S4C). However, 12–14 days after the end of BRQ treatment, mice of the BRQ group began to show xenograft growth (Fig. 4E), indicating that BRQ treatment suppressed tumor growth but did not eradicate tumor cells. Nevertheless, BRQ treatment significantly prolonged the survival of tumor-bearing mice (Fig. 4F). BRQ treatment at the indicated dose and time had no significant effect on mouse body weight (Fig. S4D).

We further evaluated the ability of BRQ to suppress the growth of established neuroblastoma xenografts. In these experiments, BE(2)-C xenografts were allowed to grow to the minimum size of 0.5 cm in any diameter (~15 days post inoculation), followed by treatment with vehicle or BRQ at 25 mg/kg via intraperitoneal injection once every other day. Again, BRQ treatment markedly slowed the growth of xenografts (Fig. 4G) and prolonged the survival of tumor-bearing mice (Fig. 4H).

Collectively, these data indicate that blocking DHODH activity can suppress the growth of *MYCN*-amplified neuroblastoma cell lines in vitro and in vivo, suggesting that DHODH is a potential therapeutic target for *MYCN*-amplified neuroblastoma.

### Physiological concentrations of uridine in the serum antagonize the action of DHODH inhibitors

**Fig. 5:**
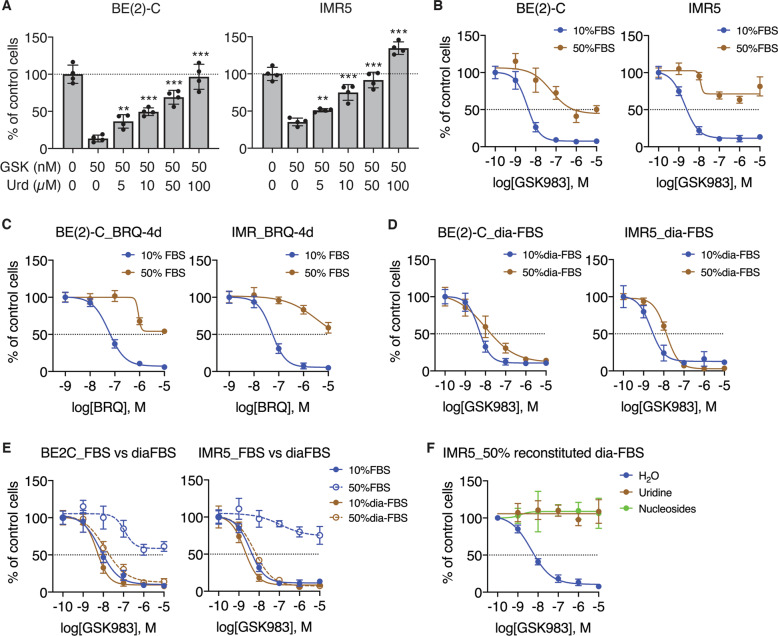
Physiological concentrations of uridine antagonize the action of DHODH inhibitors. **A** Cell-growth assay of *MYCN*-amplified cell lines treated with GSK983 in the presence of increasing concentrations of supplemental uridine (Urd) in culture media containing 10% FBS for four days. Data are mean ± SD (*n* = 4). *P* values were determined by two-tailed Student’s *t*-test between Urd-0 and individual Urd-containing groups. **P < 0.01, ***P < 0.001. **B, C** Dose–response curves for GSK983 (**B**) and BRQ (**C**) in *MYCN*-amplified cell lines cultured in the presence of 10% or 50% FBS for four days. Data are mean ± SD (*n* = 4). **D** Dose-response curves for GSK983 in *MYCN*-amplified cell lines cultured in the presence of 10% or 50% dialyzed FBS for 4 days. Data are mean ± SD (*n* = 4). **E** Dose–response curves for GSK983 in *MYCN*-amplified cell lines cultured in the presence of 10% or 50% FBS before and after dialysis for four days. Data are mean ± SD (*n* = 4). **F** Dose–response curves for GSK983 in IMR5 cells cultured in media containing 50% dialyzed FBS without or with supplemental uridine or nucleosides for four days. Data are mean ± SD (*n* = 4).

Cells can obtain pyrimidine nucleotides via both de novo synthesis and salvage (Fig. 1A), and circulating uridine is the dominant nucleoside for producing pyrimidine nucleotides via salvage [15]. The uridine concentrations in the human plasma or serum are in the range of 5–20 µM [16, 17]. We found that supplemental uridine as low as 5 µM significantly diminished the growth-inhibitory effect of GSK983 (Fig. 5A). To further assess the effect of serum uridine on the potency of DHODH inhibitors, we increased the serum content of culture media from 10% to 50% [33], based on the notion that serum constitutes ~50% of the blood. Neuroblastoma cell lines cultured in the presence of 50% FBS were highly resistant to GSK983 (Fig. 5B) and BRQ (Fig. 5C) compared with the same cell lines cultured in the presence of 10% FBS. By contrast, cells cultured in the presence of 10% or 50% dialyzed FBS were similarly sensitive to GSK983 (Fig. 5D). To eliminate other potential affecting factors, we repeated the experiments using a single bottle of FBS before and after dialysis. Again, only cells cultured in the media containing 50% FBS (before dialysis) were resistant to GSK983 (Fig. 5E). Finally, we added either uridine or a mixture of purine and pyrimidine nucleosides to the dialyzed FBS at the final concentration of 30 µM. Culture media containing 50% of the reconstituted FBS (with a final concentration of 15 µM uridine or nucleosides) completely abrogated the growth-inhibitory effect of DHODH inhibitors (Fig. 5F). Taken together, these findings suggest that serum uridine and possibly other pyrimidine nucleosides constitute an intrinsic mechanism of resistance to DHODH inhibitors.

### Blocking nucleoside transport augments the potency of DHODH inhibitors in the presence of physiological concentrations of uridine

**Fig. 6:**
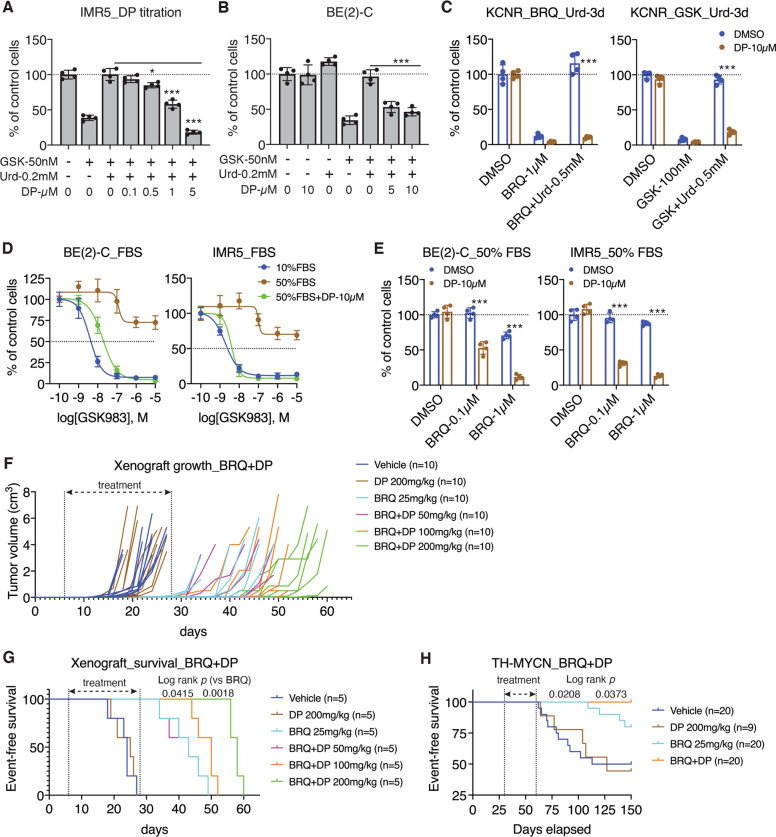
Blocking nucleoside transport abrogates uridine-dependent resistance to DHODH inhibitors. **A–C** Cell-growth assay of the indicated cell lines treated with GSK983 or BRQ without or with supplemental uridine and dipyridamole (DP) for three days. Data are mean ± SD (*n* = 4). *P* values were determined by two-tailed Student’s *t*-test. *P < 0.05, ***P < 0.001. **D** Dose–response curves for GSK983 in *MYCN*-amplified cell lines cultured in the presence of 10% or 50% FBS without or with dipyridamole for four days. Data are mean ± SD (*n* = 4). **E** Cell-growth assay of *MYCN*-amplified cell lines cultured in media containing 50% FBS for four days of treatment with BRQ in the absence or presence of dipyridamole. *P* values were determined by two-tailed Student’s *t*-test. ***P < 0.001. **F, G** Tumor-growth (**F**) and event-free survival (**G**) curves for mice bearing BE(2)-C xenografts treated with vehicle, dipyridamole (daily), BRQ (every other day), or BRQ in combination with various concentrations of dipyridamole. Log-rank test *P* values are indicated for BRQ + D*P* 100 mg/kg and BRQ + DP 200 mg/kg vs. BRQ only. **H** Event-free survival curves for *T****H****-MYCN* mice treated with vehicle, dipyridamole (daily), BRQ (every other day), or BRQ plus dipyridamole. Log-rank test *P* values are indicated for BRQ only vs. vehicle or BRQ + DP.

Mammalian cells have multiple transporters for uptake of circulating pyrimidine nucleosides [18, 19]. The above findings prompted us to test the idea that blocking uridine transport may synergize with DHODH inhibitors to suppress tumor cell proliferation in the presence of supplemental uridine. We tested dipyridamole, an FDA-approved drug (Persantine) for preventing blood clots and stroke [44, 45]. It has long been recognized that dipyridamole can block the transport of nucleosides, including uridine [46, 47]. Among the cellular targets of dipyridamole are equilibrative nucleoside transporters (ENT1–3), also known as SLC29A1–3 [48–50]. We observed a dose-dependent effect of dipyridamole in enhancing the antiproliferative activity of GSK983 in the presence of supplemental uridine (Fig. 6A), and dipyridamole at 5–10 µM completely abrogated the ability of supplemental uridine to protect cells from DHODH inhibitors (Fig. 6A–C). The addition of dipyridamole alone had no detectable effect on cell proliferation (Fig. 6B, C). Importantly, dipyridamole treatment fully restored the sensitivity of neuroblastoma cell lines to DHODH inhibitors in the presence of 50% FBS, including GSK983 (Fig. 6D), BRQ (Fig. 6E), and leflunomide (Fig. S5A). However, when cells were cultured in the presence of 50% dialyzed FBS, no such effect of dipyridamole was observed (Fig. S5B). For neuroblastoma cells cultured in the presence of 10% FBS, dipyridamole modestly enhanced the antiproliferative effect of GSK983 (Fig. S5C). Together, these findings suggest that dipyridamole-sensitive nucleoside transporters are primarily responsible for uridine uptake in neuroblastoma cells and that blocking the transport can overcome serum uridine-dependent resistance to DHODH inhibitors.

A key implication of the above findings is that targeting nucleoside transport could be a strategy for enhancing the therapeutic efficacy of DHODH inhibitors in vivo. We first tested this hypothesis in the BE(2)-C xenograft model. Tumor-bearing mice were randomly divided into six groups and treated via intraperitoneal injection with vehicle, dipyridamole at 200 mg/kg once daily, BRQ at 25 mg/kg once every other day, or BRQ plus dipyridamole at 50, 100, or 200 mg/kg. The treatment began on day 6 and ended on day 28 post inoculation (when all the vehicle-control mice were sacrificed due to tumor progression). In line with the results of the above cell-based studies, dipyridamole alone had no effect, and BRQ alone significantly slowed the growth of xenografts and extended the survival of tumor-bearing mice (Fig. 6F, G). For combination treatment, dipyridamole at 50 mg/kg showed no synergistic effect but at 100–200 mg/kg significantly boosted the efficacy of BRQ, resulting in slower growth of xenografts and longer survival of tumor-bearing mice compared with BRQ treatment alone (Fig. 6F, G). Dipyridamole at 200 mg/kg alone had no significant effect on mouse body weight and a combination of BRQ and dipyridamole at 200 mg/kg caused a modest reduction in the body weight (~7%) (Fig. S5D).

Finally, we tested the combination therapy in *TH-MYCN* mice, a transgenic mouse model of high-risk neuroblastoma with *MYCN* amplification [51–53]. *TH*-*MYCN* mice were randomly assigned to four groups and treated for 30 days via intraperitoneal injection with vehicle, dipyridamole at 200 mg/kg once daily, BRQ at 25 mg/kg once every other day, or BRQ plus dipyridamole. Again, dipyridamole by itself showed no significant effect, and BRQ treatment alone significantly extended the survival of *TH-MYCN* mice, which was further improved by the combination therapy (Fig. 6H).

Taken together, our results provide evidence that blocking nucleoside transport is a promising strategy to augment the efficacy of DHODH inhibitors as therapeutics for *MYCN*-amplified neuroblastoma.

## Discussion

In this report, we present evidence for transcriptional activation of nucleotide biosynthesis as part of the metabolic reprogramming by MYCN to support its activity in promoting cell growth and proliferation, which requires an increased supply of macromolecule building blocks, such as nucleotides, amino acids, and lipids. MYCN overexpression upregulates the expression of enzymes for de novo nucleotide synthesis, leading to an increase in the intracellular pools of purine and pyrimidine nucleotides. In addition, we show that MYCN is required for maintaining the expression of pyrimidine biosynthetic enzymes in *MYCN*-amplified neuroblastoma cell lines. Furthermore, we demonstrate that DHODH, a rate-limiting enzyme for pyrimidine synthesis, is a direct transcriptional target of MYCN critical for neuroblastoma cell proliferation and tumorigenicity. These findings are consistent with genetic data from neuroblastoma patients: only *MYCN*-amplified neuroblastoma tumors have significantly higher levels of DHODH mRNA expression compared with low-risk and high-risk neuroblastoma tumors without *MYCN* amplification. High DHODH expression is also associated with advanced stages of the disease and poor prognosis. Thus, both laboratory and patient data suggest an important role of DHODH in neuroblastoma development and progression.

We have recently shown that, because of MYCN-mediated metabolic reprogramming, *MYCN*-amplified tumor cells are increasingly dependent on key metabolic pathways for proliferation and survival, such as the serine–glycine–one-carbon metabolic network [9, 10]. Exploiting this dependency is a promising therapeutic strategy against *MYCN*-amplified tumors [9–11]. Our current study provides further evidence in support of this strategy by identifying DHODH as a promising therapeutic target in *MYCN*-amplified neuroblastoma. We demonstrated that genetic and pharmacologic inhibition of DHODH can significantly diminish the proliferation and tumorigenicity of *MYCN*-amplified neuroblastoma cell lines and slow the progression of established neuroblastoma xenografts. Moreover, we show that blocking DHODH activity can impede neuroblastoma development in *TH-MYCN* mice. It is important to note that DHODH-based therapy is most likely a safe approach, since the DHODH inhibitor leflunomide and its active metabolite teriflunomide have been used clinically for chronic diseases, such as rheumatoid arthritis and multiple sclerosis [54, 55].

The DHODH inhibitor BRQ was explored as a cancer-therapeutic agent with promising data from animal studies [38]. However, BRQ failed to show therapeutic efficacy in phase-I and -II clinical trials in patients with advanced stages of solid tumors, including breast, colon, lung, and prostate cancers [56–63]. A long-held and well-supported explanation for this failure is that inhibition of de novo pyrimidine synthesis could be compensated by pyrimidine salvage [64, 65]. Plasma uridine is taken up by cells via membrane nucleoside transporters [18]. Once inside, uridine is converted to UMP by uridine–cytidine kinase. In turn, UMP can serve as an initial building block to produce nucleotides for RNA and DNA synthesis [15, 66]. We found that increasing the serum content in culture media, which recapitulates the serum content of the blood, largely abolished the antiproliferative effect of DHODH inhibitors. This protective effect of serum was eliminated by dialysis and restored by supplemental uridine or nucleosides in the range of physiological concentrations (~15 µM). These findings suggest that plasma uridine (and possibly other pyrimidine nucleosides and bases) constitutes an intrinsic mechanism of resistance to DHODH inhibition.

Our investigation suggests that blocking nucleoside transport is a strategy to overcome the plasma uridine salvage-dependent resistance to DHODH inhibitors. We found that the FDA-approved drug dipyridamole, an inhibitor of the SLC29 family of nucleoside transporters [48–50], markedly enhanced the antiproliferative activity of DHODH inhibitors in *MYCN*-amplified neuroblastoma cell lines in the presence of supplemental uridine or high serum content. In xenograft and transgenic mouse models of *MYCN*-amplified neuroblastoma, dipyridamole at the doses of 100–200 mg/kg synergized with BRQ to suppress tumor growth and to extend the survival of tumor-bearing mice. Very recently (when this paper was in final preparation), Cuthbertson et al. reported that dipyridamole synergized with BRQ to inhibit the growth of colon and pancreatic cancer cell lines in culture. However, they observed no such synergistic effect in mouse xenograft studies [67]. One likely explanation is that they used a much lower dose of dipyridamole (10 mg/kg) in their xenograft studies compared with ours. As discussed above, we found no synergistic effect when dipyridamole was used at 50 mg/kg. However, dipyridamole at 100–200 mg/kg significantly boosted the efficacy of BRQ. Nevertheless, these findings together suggest that a combination of targeting DHODH and nucleoside transport is a promising treatment strategy for both pediatric and adult cancers.

The lethal dose 50 (LD_50_) for dipyridamole in mice is 700 mg/kg, with the safety limit at 400 mg/kg [68]. Consistent with the report, we found that dipyridamole at 200 mg/kg showed no detectable effect on mouse body weight. However, a combination of BRQ at 25 mg/kg and dipyridamole at 200 mg/kg caused a modest reduction (~7%) in the body weight of mice. Whether this is a common side effect of dual targeting of de novo and salvage pyrimidine synthesis is currently under investigation by examining the combination of BRQ with other nucleoside-transport inhibitors, such as Dilazep and NBMPR (S-(4-nitrobenzyl)-6-thioinosine) [18, 69].

In summary, we present experimental evidence for DHODH as a drug target in *MYCN*-amplified neuroblastoma. There has been a renewed interest in DHODH inhibitors as cancer therapeutics in light of recent laboratory findings [33–36]. Also, multiple clinical trials of DHODH inhibitors in adult myeloid malignancies are currently underway [70]. However, we are not aware of such trials in neuroblastoma and other pediatric cancers. Our findings provide a strong rationale for evaluating DHODH inhibitors as therapeutics for *MYCN*-amplified neuroblastoma, particularly in combination with dipyridamole or other inhibitors of nucleoside transport.

## Supplementary information

Supplemental Information

Table S2

Table S3

## Data Availability

The data that support the findings of this study are available from the corresponding authors upon reasonable request.
