# Supplementary material for: Therapeutic targeting of both dihydroorotate dehydrogenase and nucleoside transport in MYCN-amplified neuroblastoma
Source: Cell Death Dis. 2021 Aug 30;12(9):821. doi: 10.1038/s41419-021-04120-w (PMC8405683; doi:10.1038/s41419-021-04120-w)
Supplement: Supplementary file 1 — Supplemental Information [file 41419_2021_4120_MOESM1_ESM.docx]

**Supplementary Information**

**Therapeutic targeting of both dihydroorotate dehydrogenase and nucleoside transport in *MYCN*-amplified neuroblastoma**

Yajie Yu, Jane Ding, Shunqin Zhu, Ahmet Alptekin, Zheng Dong, Chunhong Yan, Yunhong Zha, and Han-Fei Ding

**Inventory of Supplementary Information**

1. **Figures S1-S5**
2. **Table S1**


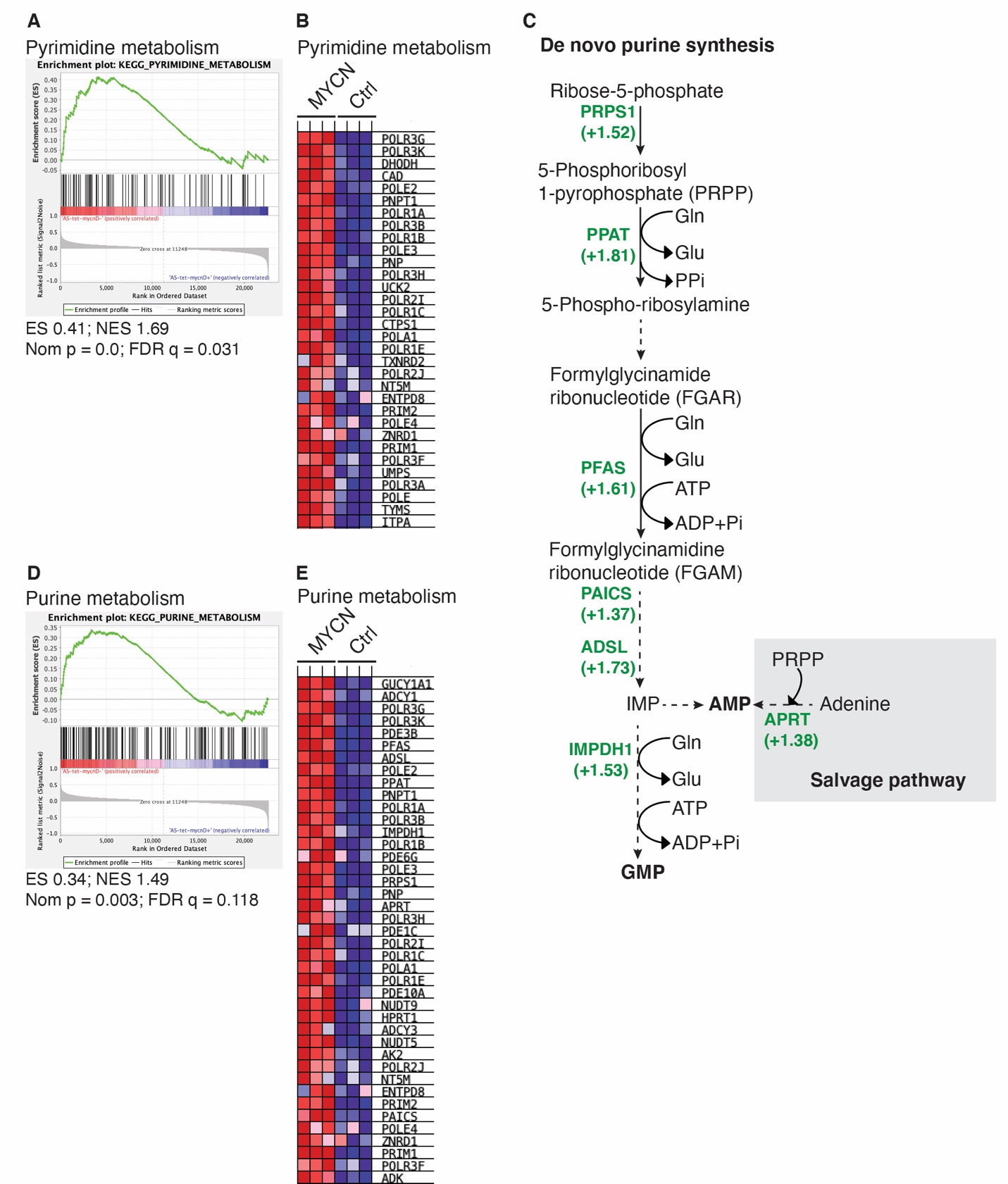


**Fig. S1.** MYCN promotes nucleotide synthesis. **A, B** GSEA of genes upregulated by MYCN showing significant enrichment of the gene set for KEGG pyrimidine metabolism. **C** Schematic of purine biosynthesis via de novo and salvage pathways with indicated fold changes in mRNA expression for pathway enzymes determined by microarray. PRPS1, phosphoribosyl pyrophosphate synthetase 1; PPAT, phosphoribosyl pyrophosphate amidotransferase; PFAS, phosphoribosyl formylglycinamidine synthase; PAICS, phosphoribosyl aminoimidazole carboxylase and phosphoribosyl aminoimidazole succinocarboxamide synthase; ADSL, adenylosuccinate lyase; IMPDH1, inosine monophosphate dehydrogenase 1; APRT, adenine phosphoribosyltransferase; Gln, glutamine; Glu, glutamic acid. **D, E** GSEA of genes upregulated by MYCN showing significant enrichment of the gene set for KEGG purine metabolism.





**Fig. S2.** High DHODH expression is associated with poor prognosis and advanced stages of neuroblastoma. **A, B** Immunoblot (**A**) and qRT-PCR (**B**) analyses of the expression of pyrimidine synthesis pathway enzymes in *MYCN*-amplified BE(2)-C and LA1-55n cells treated with DMSO (vehicle control) or the Aurora A inhibitor MLN8237 at 1.0 µM for the indicated hours (h). Data in (**B**) are mean ± SD (n = 4). *P* values were determined by two-tailed Student’s t-test. ***P < 0.001. **C** ChIP-seq profile for MYC binding to the *DHODH* locus using ENCODE transcription factor ChIP-seq data. Also indicated are histone H4 lysine 4 monomethylation and trimethylation and H3 lysine 27 acetylation marks that are associated with regulatory elements and active chromatin. **D** Kaplan-Meier survival curves for three independent cohorts of neuroblastoma patients based on DHODH mRNA expression, with log-rank test *P* values indicated. **E** Box plots of DHODH mRNA expression in relation to neuroblastoma stages using datasets from the three neuroblastoma cohorts. Data were analyzed with ANOVA (stages 2, 3, 4 vs. stage 1), with *P* values indicated.


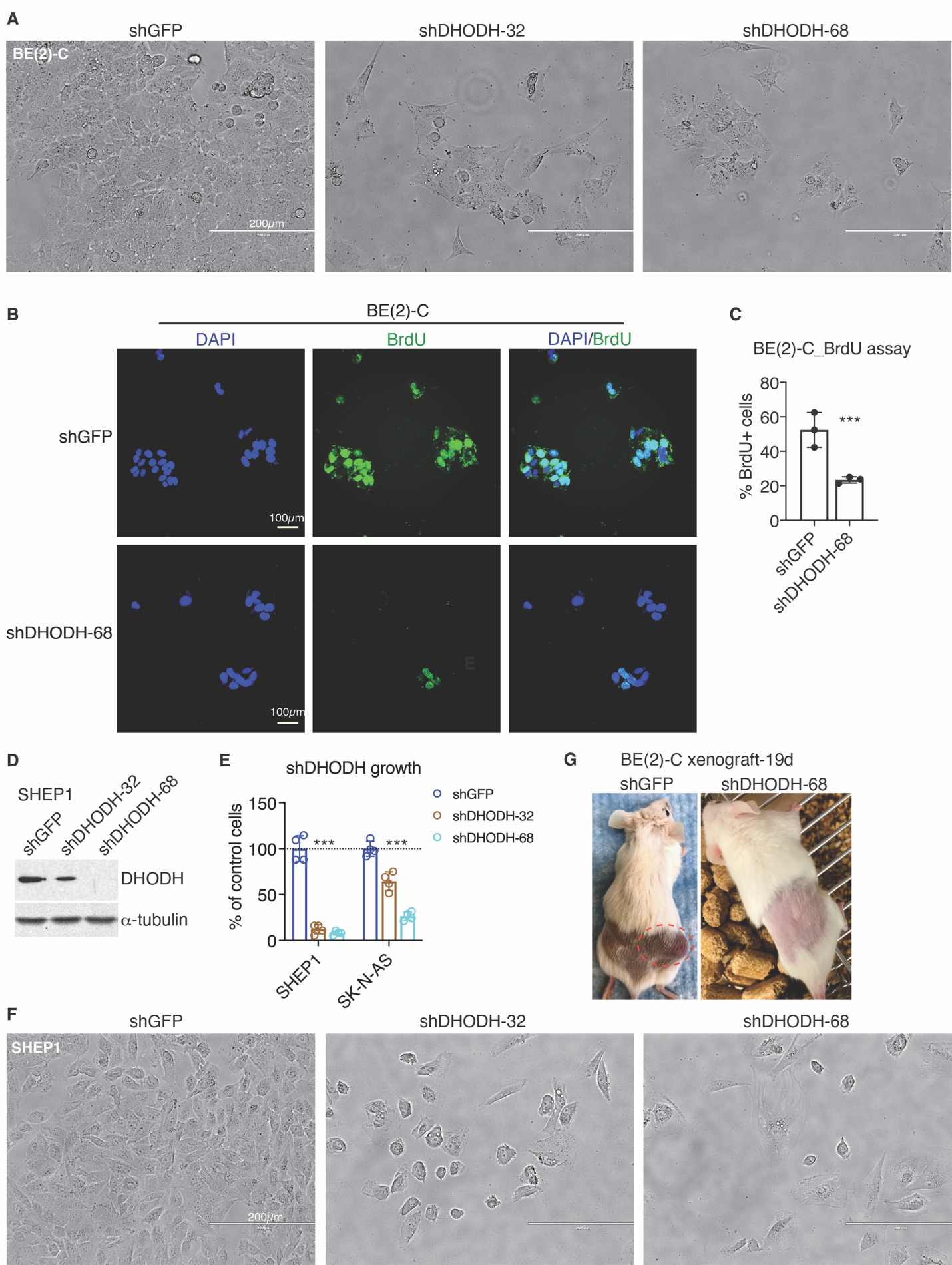


**Fig. S3.** High DHODH expression is essential for neuroblastoma cell proliferation. **A** Phase-contrast images of *MYCN*-amplified BE(2)-C cells without (shGFP) or with DHODH knockdown (shDHODH) following 4 days of culture. **B** BrdU immunofluorescence images of BE(2)-C cells without (shGFP) or with DHODH knockdown (shDHODH) following 4 days of culture. **C** Quantification of BrdU-positive BE(2)-C cells without (shGFP) or with DHODH knockdown (shDHODH) following 4 days of culture. Data are mean ± SD from three independent experiments. *P* value was determined by two-tailed Student’s t-test. ****P* < 0.001. **D** Immunoblot analysis of DHODH expression in non-*MYCN*-amplified SHEP1 expressing shRNA to GFP or DHODH. α-tubulin levels are shown as loading control. **E** Cell growth assay of non-*MYCN*-amplified cell lines without (shGFP) or with DHODH knockdown (shDHODH) for 4 days. Data are mean ± SD (n = 4). *P* values were determined by two-tailed Student’s t-test. ***P < 0.001. **F** Phase-contrast images of non-*MYCN*-amplified SHEP1 cells without (shGFP) or with DHODH knockdown (shDHODH) following 2 days of culture. **G** Images of representative mice bearing BE(2)-C xenografts (shGFP vs. shDHODH-68) on day 19 post inoculation.


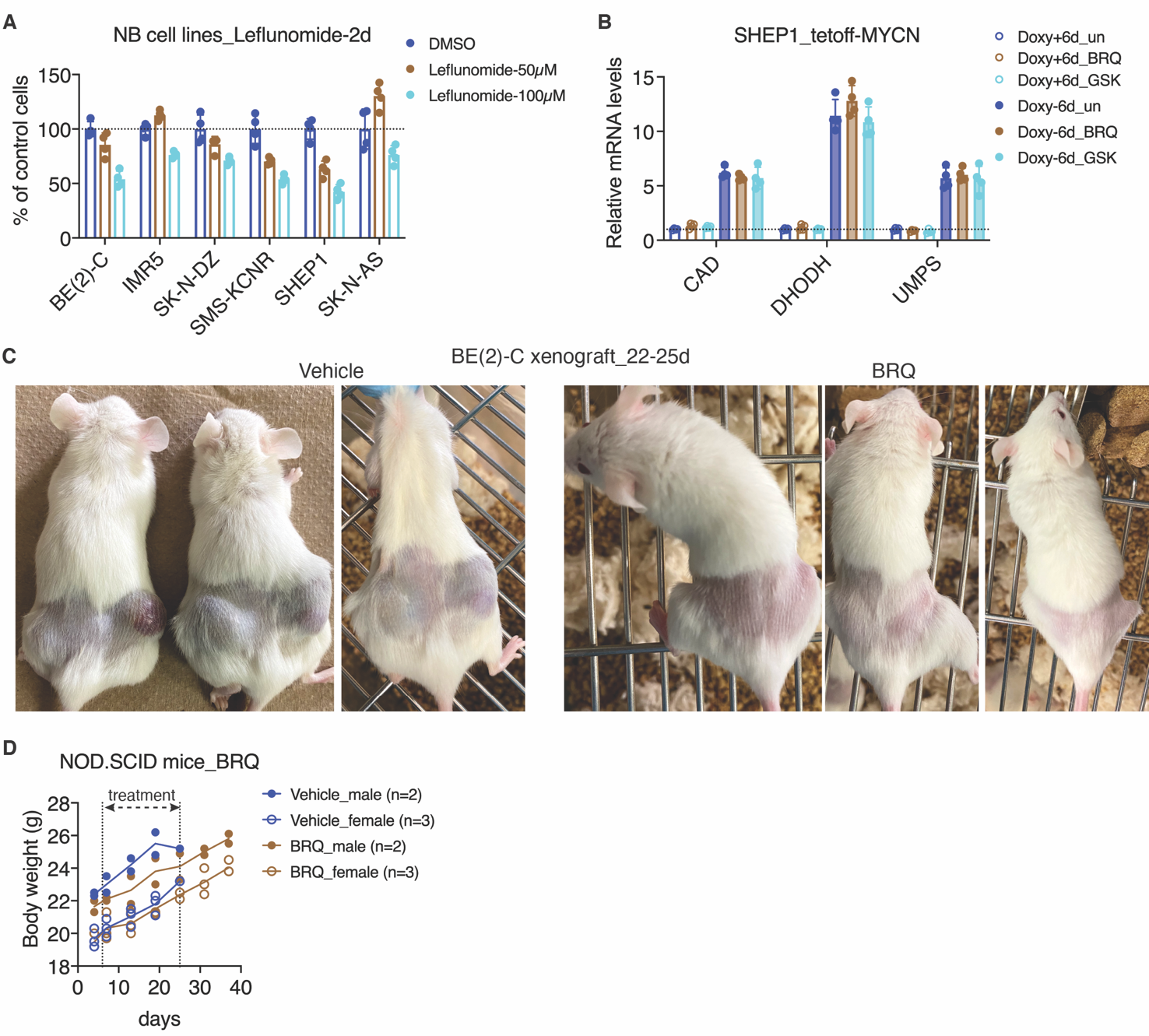


**Fig. S4.** DHODH is a therapeutic target in neuroblastoma. **A** Cell growth assay of the indicated neuroblastoma cell lines treated with DMSO or 50-100 µM of leflunomide for 2 days. **B** qRT-PCR analysis of mRNA expression for pyrimidine synthesis enzymes in non-*MYCN*-amplified SHEP1 cells without or with MYCN induction in the absence of doxycycline (Doxy-) for 6 days. Cells were treated with DMSO (un), 1.0 µM BRQ or 100 nM GSK983 for 2 days. Data are mean ± SD (n = 4). **C** Images of representative mice bearing BE(2)-C xenografts on day 25 post inoculation. Mice were treated with vehicle or BRQ at 50 mg/kg every 3 days. **D** Body weights of xenograft-bearing mice treated with vehicle or BRQ at 50 mg/kg every 3 days.


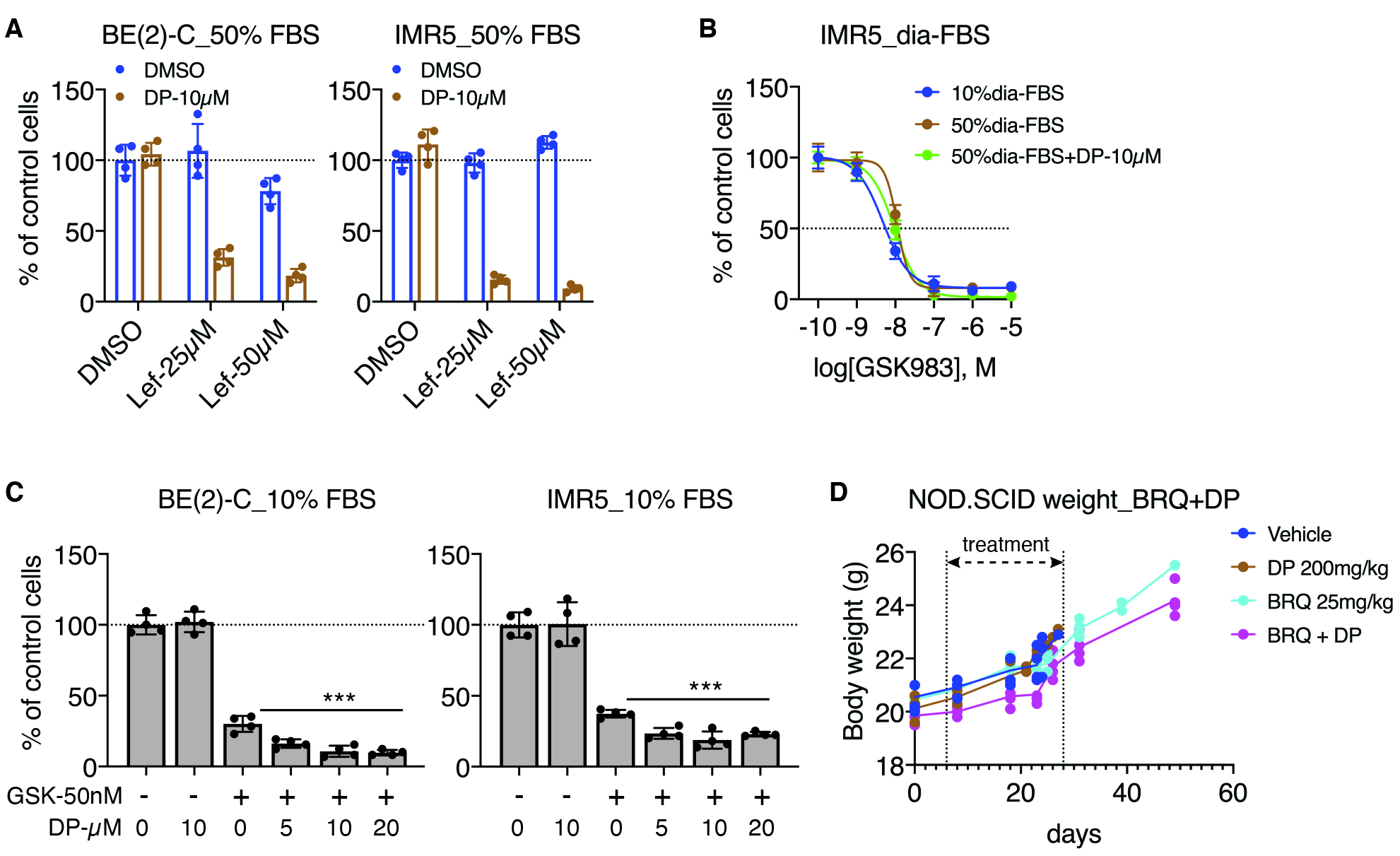


**Fig. S5.** Blocking nucleoside transport abrogates serum uridine-dependent resistance to DHODH inhibitors. **A** Cell growth assay of *MYCN*-amplified cell lines cultured in media containing 50% FBS following 4 days of treatment with leflunomide (Lef) in the absence or presence of dipyridamole (DP). *P* values were determined by two-tailed Student’s t-test. ****P* < 0.001. **B** Dose-response curves for GSK983 in IMR5 cells cultured in the presence of 10% or 50% dialyzed FBS without or with dipyridamole for 4 days of treatment. Data are mean ± SD (n = 4). **C** Cell growth assay of *MYCN*-amplified cell lines cultured in media containing 10% FBS following 4 days of treatment with GSK983 in the absence or presence of dipyridamole. *P* values were determined by two-tailed Student’s t-test. ****P* < 0.001. **D** Body weights of xenograft-bearing mice treated with vehicle, dipyridamole (daily), BRQ (every other day), or BRQ plus dipyridamole.

| **Table S1. qRT-PCR primers** | | |
| --- | --- | --- |
| **Primer set** | **Forward 5’-3’** | **Reverse 5’-3’** |
| ATIC | ACCTGACCGCTCTTGGTTTG | TACGAGCTAGGATTCCAGCAT |
| CAD | TAGTCCTTGGCTCTGGCGTCTA | TAGTCGGTGCTGACTGTCTCTG |
| DHODH | GCAAAGTCAAGCGGGAACTG | TCCAATGGCATCTGTGACTCC |
| MYCN | ACCACAAGGCCCTCAGTACCTC | TGACAGCCTTGGTGTTGGAGGA |
| PAICS | TTGCAGAAGAATAGCAACTGGTT | CACTGTGGGTCATTATTGGCAT |
| PFAS | CCCAGTCCTTCACTTCTATGTTC | GTAGCACAGTTCAGTCTCGAC |
| PPAT | GATGGGAGTTCGGTGCCAA | CAACGAAGGGCTGACAATTTTC |
| PSPH | GACAGCACGGTCATCAGAGAAG | CGCTCTGTGAGAGCAGCTTTGA |
| UMPS | GCCTCCTTATTGCGGAAATGAGC | CTGGTTTCATGCTTACTCGGGAG |
| B2M | TGCTGTCTCCATGTTTGATGTATCT | TCTCTGCTCCCCACCTCTAAGT |
